# How to use participatory design to develop an eHealth intervention to reduce preprocedural stress and anxiety among children visiting the hospital: The Hospital Hero app multi-study and pilot report

**DOI:** 10.3389/fped.2023.1132639

**Published:** 2023-02-14

**Authors:** Charlotte C. Poot, Eline Meijer, Annet Bruil, Melanie Venema, Niko J. H. Vegt, Nicole Donkel, Veronique van Noort, Niels H. Chavannes, Arno A. W. Roest

**Affiliations:** ^1^ Department of Public Health and Primary Care, Leiden University Medical Centre, Leiden, Netherlands; ^2^ National EHealth Living Lab (NeLL), Leiden University Medical Centre, Leiden, Netherlands; ^3^ Design for Impact, Rotterdam, Netherlands; ^4^ The Willem-Alexander Pediatric Hospital, Leiden University Medical Centre, Leiden, Netherlands; ^5^ Faculty of Industrial Design Engineering, Delft University of Technology, Delft, Netherlands

**Keywords:** anxiety, coping, eHealth, procedural comfort, participatory design (PD), co-creation

## Abstract

**Background:**

Medical procedures can cause considerable stress and anxiety among children. Current interventions mainly diminish stress and anxiety during procedures, while stress and anxiety often build up at home. Moreover, interventions often focus on either distraction or preparation. eHealth can combine multiple strategies and provide a low-cost solution that can be used outside the hospital.

**Objective:**

To develop an eHealth solution to diminish preprocedural stress and anxiety, and to evaluate the app on use, usability and user experience in practice. We also aimed to gain in-depth insights in children's and caregivers' opinions and experiences to inform future improvements.

**Methods:**

This is a multi-study report on the development (Study 1) and evaluation (Study 2) of a first version of the developed app. In study 1 we adopted a participatory design approach in which children's experiences were central to the design process. We performed an experience journey session with stakeholders (*n* = 13) to map the child's outpatient journey, identify pains and gains, and formulate the desired experience journey. Iterative development and testing with children (*n* = 8) and caregivers (*n* = 6) resulted in a working prototype. The prototype was tested with children, resulting in a first version of the Hospital Hero app. The app was evaluated on use, user-experience and usability during an eight-week pilot study in practice (Study 2). We triangulated data from online interviews with children and caregivers (*n* = 21) and online questionnaires (*n* = 46).

**Results:**

Multiple stress and anxiety experience touchpoints were identified. The Hospital Hero app supports children in their hospital journey by facilitating preparation at home and providing distraction at the hospital. The pilot study showed that the app was evaluated positively on usability and user-experience and is considered feasible. Qualitative data showed five themes: (1) user-friendliness, (2) coherence and power of storytelling, (3) motivation and reward, (4) fit with real hospital journey, (5) procedural comfort.

**Discussion:**

Using participatory design, we developed a child-centered solution that supports children in the entire hospital journey and may diminish preprocedural stress and anxiety. Future efforts should create a more tailored journey, define an optimal engagement window and formulate implementation strategies.

## 1. Background

### 1.1. (Pre)procedural anxiety and its negative (health)consequence in children—short and long term

Medical procedures in children such as blood drawing performed at the outpatient department are often accompanied by stress and anxiety before and during the procedure (1). Feelings of stress and anxiety during procedures may elicit strong behavioral responses such as crying, withdrawal or showing uncooperative behavior. Subsequently, procedural distress, especially experienced in early life, can result in numerous disadvantageous psychological health outcomes (2) and pose a substantial burden on children, their caregivers and healthcare providers involved. Generally, children are able to remember their experiences and exaggerate these negative memories when considerable distress is experienced, which in turn can lead to more distress in subsequent hospital visits (3). In the long term, procedural distress can have severe implications for the individual experiencing distress and the society as a whole.

A growing body of evidence shows that early childhood hospital-related trauma is associated with hospital-induced anxiety and needle trauma later in life (4–6). Also negative experiences with healthcare may negatively influence a child's attitude towards healthcare and healthcare providers in general and influence appropriate coping skills in adulthood (6). In addition, distress may increase pain sensation and decrease analgesic efficacy, resulting in a higher titration need and decreased compliance with future procedures and treatments (7).

### 1.2. Coping strategies

While invasive procedures such as blood drawing or injections are a common source of preprocedural distress, non-invasive procedures or the hospital environment in general can also cause stress and anxiety (6). As such, procedural comfort, directed at minimizing distress throughout a hospital visit, is considered an essential part of pediatric care and has been included in medical guidelines as a necessary adjuvant to procedural sedation. In fact, more recently, academics have opted for procedural comfort as the starting point and sedation as the adjuvant. Procedural comfort aims to provide children with appropriate coping skills. As such, these non-pharmacological interventions can roughly be categorized into (1) distraction (i.e., diverting the attention from the procedure to something more positive; e.g., focus on an object, watching a funny movie), (2) emotional control (e.g., use of comfort talk, relaxation), (3) psychological preparation (i.e., information about procedures and sensations to expect, normalization of anxiety) and giving the child a feeling of autonomy and control. Providing children with coping skills is especially important for younger children, as they are not yet able to verbally express their feelings and understand the rationale for a specific procedure as a way to cope with a stressful event (8). Additionally, the child's understanding of the degree of discomfort expected is not well developed.

### 1.3. Limitations of current interventions

While studies show that the above-mentioned coping strategies help to alleviate the pain, stress and anxiety, this relief is often only effective at the moment and thus temporary. Moreover, no single intervention is equally effective on its own. Hence optimal procedural comfort asks for a combination of strategies. However, these multi-strategy interventions also require skilled and trained staff and a favorable family-centered environment. Unfortunately, this is far from reality within daily procedural care with departments facing fast patient turnover, high workload, limited time to explain procedures and limited budget for the training of all the staff (11).

Another limitation is the fact that current efforts mainly focus on reducing stress and anxiety experienced within the consultation or treatment room or relatively shortly beforehand (e.g., explain what is going to happen). However, stress and anxiety often build up already at home in anticipation of the visit and the pain induced by the procedure, and can peak during the procedure, causing the child to be distressed and upset. Hence, minimizing this so called prehospital and preprocedural stress and anxiety is important to alleviate feelings of stress and anxiety during the visit. Psychological and educational preparation is an important way to achieve this and happens in an out-patient setting through information leaflets or online information. However, most information and research on the impact of proper preparation is focused on children undergoing surgery (9, 10).

Moreover, the available information resources often lack child-centered information, are not easily accessible, are dispersed and vary in quality (11). In addition, there seems to be a mismatch between the current provision of procedural information and children's and caregivers' expectations that information will be provided directly to them by healthcare professionals (12). This mismatch is strengthened by the fact that caregivers may, in their gatekeeping role, limit access to preparation materials and thereby unwillingly disempower their child (11, 13). Hence, interventions aimed at reducing preprocedural stress and anxiety should be child-centered, combining multiple effective strategies and directed at diminishing stress and anxiety already at home.

### 1.4. Leveraging potentials of eHealth

Digital health technologies such as (health) apps offer the potential to provide a low-cost solution outside of the hospital setting. In addition, multiple procedural comfort strategies can be combined and offered in a way that does not require additional training or disruptions of daily workflows of hospital staff. As such, apps can create engaging, interactive child-appropriate content, including elements of play that can be delivered and accessed in a time-appropriate manner and within the comfort of the child's own home. Innovations that leverage the potentials of digital health technologies such as “Xploro (i.e., a platform that provides child-centered healthcare using gameplay and artificial intelligence) demonstrated that procedural knowledge is improved and levels of self-reported anxiety are reduced (14). Also, virtual reality has been shown to be promising in reducing distress during painful procedures (15, 16). Nonetheless, these interventions either have a single purpose (either preparation or distraction), are situated within the hospital, or require training of staff.

### 1.5. Design and implementation of eHealth

Despite its potential, digital health technologies are abundant in number but only sparsely implemented as standard care. Reasons for lack of uptake include issues in usability (i.e., ease of use, task performance using the app), poor integration within current healthcare workflows and habits, and issues regarding the value it brings to the user (e.g., making a task more efficient, or more pleasant) (17). To stimulate uptake and usage, apps need to fit the user's needs and daily lives, be considered useful and user friendly. Involving children and caregivers early in the design and development is paramount in ensuring that the app is child-centered and fits children's daily life and experience world. In addition, stakeholders need to be involved in the design process to ensure optimal uptake in practice and fit with everyday healthcare practices.

### 1.6. Participatory service design

One way to accomplish this is by applying a Participatory Design approach (PD) in combination with a service design approach. PD is a methodology that promotes the participation of users and other stakeholders in the design of technology, such as apps, by involving them in several phases during the design process (18). Service design adds by taking the experience journey as a starting point, including varying processes, experiences and people who contribute herein (19). PD can be divided into four phases: the identification of users' needs (phase 1, discover); the generation of ideas and development of prototypes and testing (phase 2, prototype); realization (phase 3) and evaluation (phase 4). PD can be seen as an iterative process where each phase is planned by reflecting on the results of the previous phase with respect to the participants’ contributions. The iterations ultimately result in a first version of a digital health technology or service (minimum viable product; MVP) that can be evaluated in practice. Evaluation in practice is important to gain in-depth insight into actual use of the product or service, its usability, and how the product or service is used (user-experience), and to identify improvements and inform further implementation and scale-up.

#### 1.6.1. Study objectives

The objective of this study was two-fold. First, following a PD approach, we aimed to develop an eHealth solution to reduce preprocedural stress and anxiety among children visiting the hospital's outpatient clinic. The second objective was to evaluate the app on use, user experience and usability in practice and to gain in-depth insight into the opinions and experiences of children and their caregivers to inform future improvements.

#### 1.6.2. Method

##### 1.6.2.1. Study design

**Figure 1:**
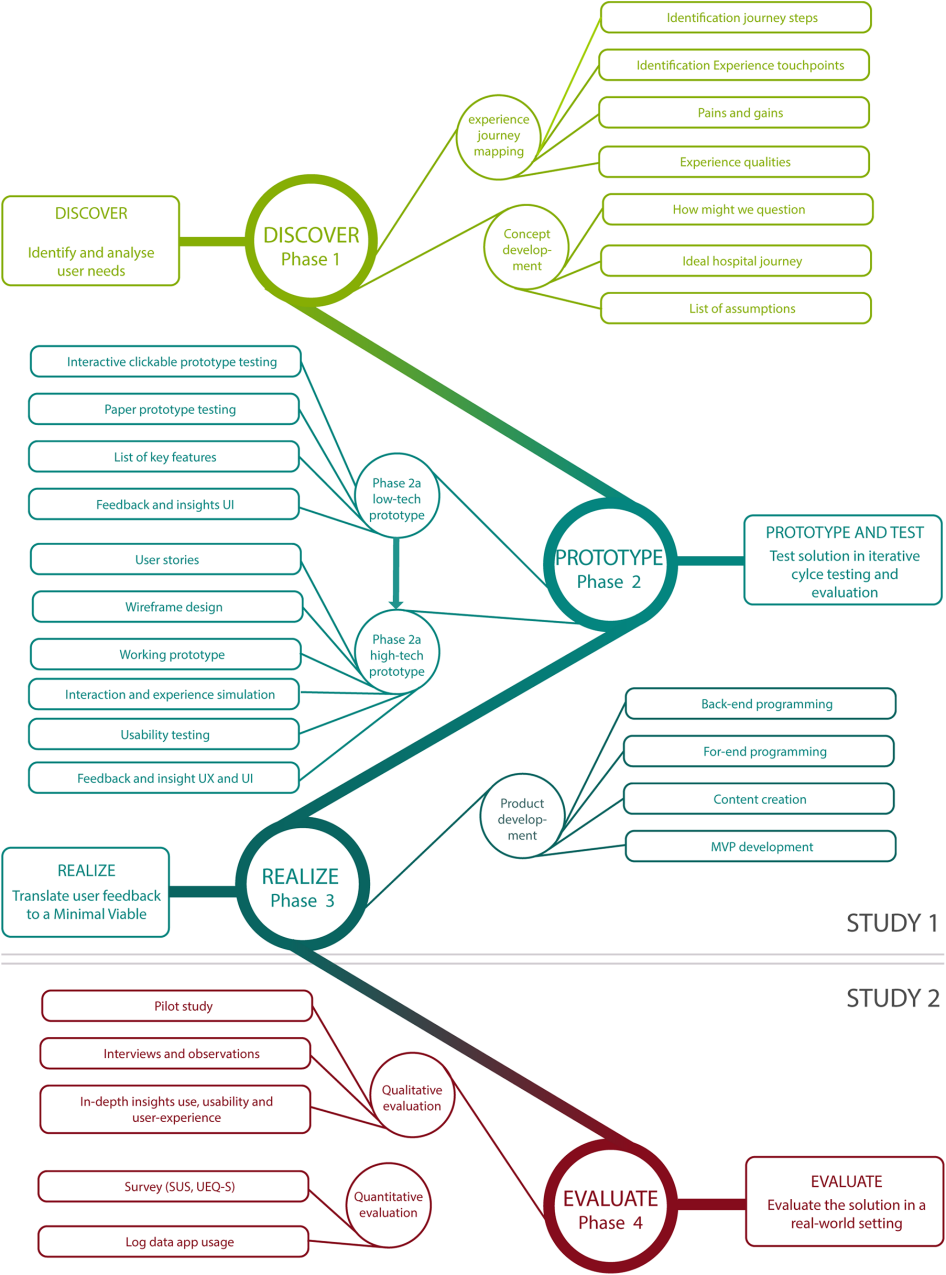
Schematic overview of study design of study 1 and study 2, mapped onto participatory design phases. UX, user experience; UI, user interactions; MVP, Minimum Viable Product; SUS, System Usability Scale; UEQ_S, User Experience Questionnaire Short Version.

In this paper, we report on two studies performed to develop and evaluate the eHealth solution. The first study describes the development process that led to a first version of an application. The process can be roughly divided into three phases, corresponding with the first three PD phases. In phase 1, users-needs were identified. Phase 2 consisted of two iterations during which prototypes were developed, refined and tested together with children. In phase 3, results from the final prototype testing were used to realize a first version of the app. The second study describes the evaluation study in the form of a pilot study at the outpatient clinic. The study corresponds with the fourth and final PD phase and closes with recommendations for improvement and future implementation. See Figure 1 for a schematic representation of the study design.

## 2. Study 1

### 2.1. Method

#### 2.1.1. Design and context

The study followed the first three phases of the participatory design cycle. It was conducted between February 2020 and July 2020 in the outpatient clinic of the academic pediatric hospital in Leiden, the Netherlands. An initial idea, initiated by two pediatric nurses, formed the starting point of this study. The idea, given the name Hospital Hero, included elements of distraction, game play and an animal or hero theme.

#### 2.1.2. Participants and procedures

##### 2.1.2.1. Phase 1: discover

An experience journey session was held with stakeholders to identify user needs and potential touchpoints where a stress-reducing app would be of value (20). Stakeholders were those involved in pediatric outpatient care (e.g., pediatric doctor, pediatric nurse, child psychologist, doctor's assistant) and those important for the realization of the app and content developers (i.e., app developers, educational content development experts, eHealth experts). Considering the explorative nature of the session, a patient advocate youngster was invited to participate in this early stage instead of young children. The experience journey session was facilitated by an experienced participatory (service) designer (author AB) and consisted of three parts. First, the separate process steps of a visit to the outpatient department were mapped out, including the moments at home and to and from the hospital. Second, experience touchpoints from the child, caregiver and professional were identified, mapped on the journey map and used to identity critical moments within the journey. Experience touchpoints included “pains” (moments that contribute to (pre)procedural stress and anxiety) and “gains” (moments where design could alleviate the stress and anxiety). Third, pains and gains were used to identify design opportunities and ideas for solutions and experience qualities (i.e., properties the designed service or product must have to fulfil the user's needs in terms of desired experience) were brought in by all participants using the “ how might we” question technique. A final voting round on the most important experience qualities resulted in a selection of properties that the concept should embody and that led to the ideal journey.

##### 2.1.2.2. Phase 2: prototype—iteration 1

A multi-disciplinary development team was installed, consisting of two pediatric nurses, a pediatric doctor, an eHealth expert, an app developer and a user experience designer. This team translated experience qualities into a concept for the app and low-cost clickable prototype and accompanying paper prototypes. This approach ensured that the concept fits with the users' needs and could be convincingly communicated to all stakeholders while circumventing full development costs. The prototypes embodied the essential user interactions. Assumptions on user interaction and user experience were formulated *a priori*. The prototypes and the assumptions were tested with children and caregivers at the outpatient clinic of the Willem-Alexander Children's Hospital in Leiden, the Netherlands, during two observation days. Children who had an appointment during the testing days were selected following purposive sampling to ensure a variation in gender, age (between 4 and 10 years old), type of visit and prior hospital experience. A letter was sent to all participants two days prior to the observation day to test the entire concept, including the process taking place at home (i.e., informing children and caregivers about the app, preparing for the visit with the app), and provide a realistic experience. The letter included a QR code with which the participants could download the app and a form on which caregivers could indicate their interest to participate in the study. Two of the eight invited caregivers indicated that their child did not experience stress or anxiety. In total, six caregivers and eight children participated in the prototype testing day. This number was deemed sufficient to test the assumptions (21). During the prototype testing day, observations were held, followed by a short semi-structured interview with the child and caregiver. The interviews were guided by a topic list covering the *a priori* assumptions.

##### 2.1.2.3. Phase 2: prototype—iteration 2

Findings from the prototype testing were discussed with the development team. A list of necessary features was drafted and translated into user stories (i.e., stories describing the needed functionality from a user's perspective) which were used to guide and prioritize the development process. A working prototype was developed, which included essential features for the app and acted as a real app (e.g., user interaction, navigation, visual designs). Due to covid 19-measures, the prototype could not be tested at the outpatient clinic. Instead, a hospital setting was built in an external setting where a hospital visit was simulated (e.g., a waiting room, consultation room). Children from the development team's social network were invited for the simulation testing day. The aim of the test was to evaluate user-interactions with the app (e.g., can the user navigate through the app, are there functionalities the user does not understand) and basic user-experiences (e.g., does the user enjoy key activities in the app such as selecting a favorite animal, what does the user like/dislike). Therefore, it was not necessary that the children visited the outpatient clinic and/or had any hospital experience. Two developers observed how the participants performed. An observation list was used to take notes on users' errors/problems and users' expressions for each task. Findings were discussed with the development team and used to identify necessary features and improvements.

##### 2.1.2.4. Phase 3: realize

Improvements were made and the app's functionalities and design were further refined based on input from the development team. This resulted in a first version (minimum viable product, MVP) of the Hospital Hero app ready to be pilot-tested and evaluated further in practice.

#### 2.1.3. Ethical considerations

Informed consent from all participants was given prior to study activities. Ethical review and approval was not required for the study on human participants in accordance with the local legislation and institutional requirements. Written informed consent to participate in this study was provided by all participations prior to study activities. In the case of children, informed consent was provided by the participants' legal guardian/next of kin.

### 2.2. Results

#### 2.2.1. Phase 1: discover

**Figure 2:**
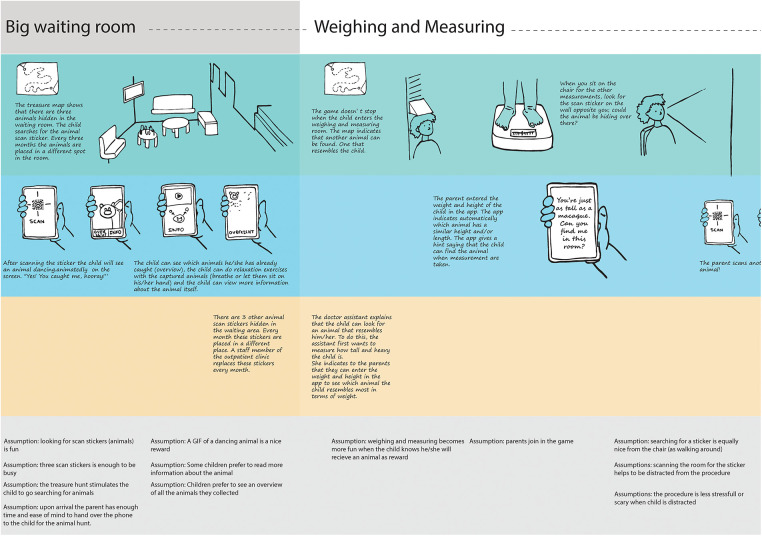
Part of the hospital Hero concept journey including the desired experience qualities and design assumptions.

The experience journey mapping session (identifying the process steps and experience touchpoints) resulted in a visual representation of the experience journey with ten distinct steps:(1) At home; (2) to the hospital; (3) at hospital registration; (4) at the outpatient registration desk; (5) In the big waiting room; (6) Weighing and measuring; (7) In the consultation room; (8) Blood drawing; (9) Leaving the hospital; (10) Back home. There were multiple (potential) stress and anxiety experience touchpoints, although it became apparent that touchpoints differed strongly between children, possibly due to character traits or prior experience. In general most prominent stress and anxiety experience touchpoints were when entering the building, in the waiting room, taking of the clothes for weighing or physical examination, “giving” their arm for blood drawing, and seeing the needle or attributes (e.g., arm cuff) associated with prior (painful) experiences. Healthcare professionals also noted that caregivers might unwillingly project their stress on the child. As blood drawing is the most common invasive procedure, it was decided to focus on reducing stress and anxiety surrounding blood drawing visits. Finally, the most important experience qualities (e.g., child should be distracted during waiting, app should be engaging during multiple visits, child should be in charge) were identified and resulted in a description of the ideal hospital journey for children with the desired experience qualities (see Figure 2).

#### 2.2.2. Phase 2: iteration 1

##### 2.2.2.1. The hospital Hero concept

The desired experience qualities, such as children are distracted searching, were translated into the Hospital Hero concept. The Hospital Hero concept is based on three core elements: (1) preparation, (2) distraction and (3) supporting caregivers in supporting their children. The Hospital Hero app supports and guides children in their journey through the hospital using a “ discovery map” in the app. The map visualizes the different rooms in the outpatient clinic (the waiting room, weigh and measure room, consultation room, blood drawing room). It also includes the steps at home, going to and leaving the hospital. Children can download the app already at home and watch short videos in the different steps together with their caregiver, such that they become informed about the different procedures (preparation and parental support). Children can search for and collect animals in the hospital by scanning QR codes (distraction). Every three months, the children can search for new animals, found at new hiding places to keep the app engaging over time. The concept was visualized into the Hospital Hero journey scheme. For each step in the journey, the scheme included a detailed description of the desired interaction moments between child and app, child and caregiver, and child and healthcare provider, as well as a set of assumptions (see Figure 2 for an exert of the concept Hospital Hero journey and Supplementary File 1 for the full concept and assumptions).

##### 2.2.2.2. Low-fidelity prototype testing

To test the assumptions and the Hospital Hero concept as a whole, the concept was translated into a clickable prototype of the app, including a schematic representation of the different steps of an outpatient visit and a short animation video on blood drawing. In addition, a paper-prototype version of the discovery map was created that simulated the animal collection game and experience. Children could use the paper discovery map to collect animal stickers. Prototype testing showed that children enjoyed looking for animals and collecting animals and that it distracted them while they were waiting. Searching for animals was less suitable in the physicians' consultation room and could be disturbing. Caregivers rather focused on the conversation with the physician. Due to logistical difficulties, not all caregivers had received the invitation letter with the link to download the app, so they had not used it in preparation for the visit. Caregivers however did indicate that they thought it could help them and their child to prepare for the visit by for example knowing what to say. Caregivers who received the letter did not feel that it addressed their needs (reducing stress and anxiety) as their child was not anxious. They suggested to include the fun and play element in the invitation letter as that was considered of value to them.

#### 2.2.3. Phase 2: iteration 2

Prototype testing resulted in several key functionalities which were translated into user stories (see Table 1) and wireframes that visualized the flow of the app. Both user stories and wireframes were discussed with the development team and used to develop a first working prototype. The prototype was tested with children on usability, user-interaction (e.g., does the child understand how to collect an animal, can the child navigate to the animal collection) and basic user experience (e.g., does the child enjoy collecting the animal, selecting a favorite animal) in a hospital simulation setting. Overall, children understood how to scan a QR code and collect an animal. Younger children (<6 years) needed additional instructions and an adult who demonstrated the scanning process, but they were able to continue playing the app afterward. Children liked searching for animals, and enjoyed receiving the animation of a dancing monkey as a reward token when all animals were found. Children noted that the “reward tune” could give away the animal's location to other children. On the other hand, it also encouraged children to search together. We observed that children missed feedback regarding the number of animals that could be found and that the majority of the children did not read the text beneath the animal picture presenting fun facts about the animal. The user test results were discussed with the development team and resulted in three important improvements: (1) users should receive a small reward after each animal found, (2) the screen with animal facts should be less text-heavy with bigger and more pictures and (3) users should be able to see how many animals can be found per step.

**Table 1:** List of key functionalities and user stories of the hospital Hero app.

Key functionalities	User stories
Onboarding	Onboarding screens explain the setup of the app (journey map and hidden animals) to the child in visual way.
Journey map	Children can see the separate steps of the (anticipated) hospital journey and navigate between them.
Information per step	Children can read information on what to expect at each step in the map and see a picture of the room or another relevant image.
Preparation video	Children and caregivers have the option to watch a short animation video explaining what happens during the blood drawing procedure, what the child can expect and certain preference they may have (e.g. sit on caregivers lap or alone).
Animal search game	Children can collect animals by scanning QR codes which are hidden in the waiting room, in the room where the child is measured, in the consultation room and in the blood drawing room
	Children can scan a QR code using the smartphone’s camera. Children hear a happy tune when the animal is collected. The animal is automatically added to their animal collection
Animal collection	Children can access their animal collection any time and read fun facts about the animals they collected. A silhouette indicates that the animal still needs to be found.
Favorite animal game	Children can select their favorite animal from a list of animals. The favorite animal joins them on their journey through the hospital.
General information about the app	Caregivers can read general information about the Hospital Hero app, Hospital Hero’s mission and the privacy policy

#### 2.2.4. Phase 3: realize

**Figure 3:**
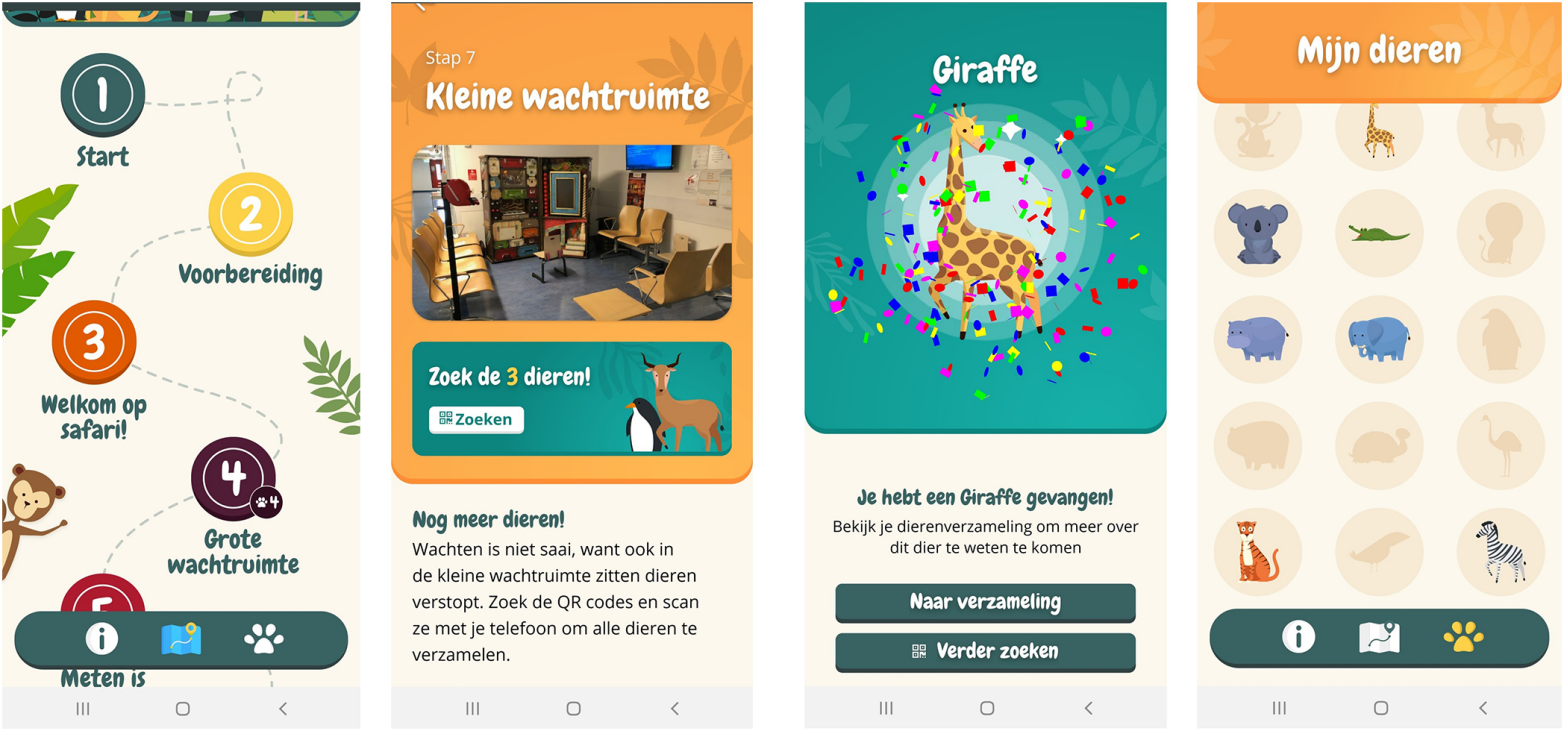
Visuals of the final version of the hospital Hero app. From left to right: “Journey map” homescreen, waiting room step, new animal collected, animal collection overview.

To use resources efficiently, it was decided to only develop the app for Android in this phase of the project. To ensure efficient development of the iOS version, it was ensured that all functionalities were equally compatible for iOS. Final refinements were made, resulting in a first version of the Hospital Hero app (minimum viable product, MVP). The final content of the app was drafted with experts on comfort talk. See Figure 3 for a visual presentation of the key features and designs of the Hospital Hero app. Parallel to the app development, a content management system was developed that could, in the future, be used by the hospital staff themselves to manage the app's content and thereby tailor it to their own hospital context.

## 3. Study 2—pilot study

### 3.1. Method pilot study

#### 3.1.1. Study design and theoretical framework

The first version of the Hospital Hero app was pilot-tested and evaluated during an eight weeks pilot study at the outpatient clinic of an academic pediatric hospital in Leiden, the Netherlands. We conducted a prospective observational study using a concurrent mixed-method approach (i.e., simultaneous collection of qualitative and quantitative data) to gain in-depth insight and provide generalizable results to inform future use. Data was collected in January and February 2021. Quantitative data were collected through an online questionnaire. Observations, online semi-structured interviews and open questions in the online questionnaire were used to collect qualitative data.

**Figure 4:**
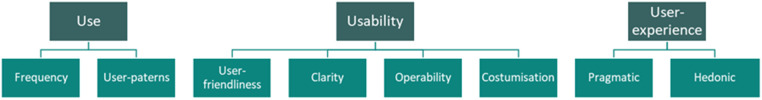
Theoretic framework on use, usability and user-experience.

Our study design, research materials and analyses were inspired by the ISO norms for “ Systems and software Quality Requirements and Evaluation; ISO/IEC 9126-1”. Key constructs were usability and user experience. The construct usability can be defined as “ the capability of the software product to be understood, learned, used and satisfying to use”. As such, it can be subdivided into user- friendliness (i.e., degree to which the software is adapted to the skills and experience of the user) clarity (i.e., the degree to which the software is considered coherent, logical and consistent) operability (i.e., the time it takes the user to learn how to use a function and perform a task efficiently) and customization (i.e., the degree to which the software system can be customized to the needs of the user (e.g., default settings). The construct user-experience was defined as “every emotion, belief, perception, psychological reactions and behavior during and after the use of a product” (22). As such, user-experience can be categorized into pragmatic experiences (i.e., task oriented aspects such as efficiency, learnability etc.) and hedonic experiences (i.e., non-task oriented aspect such as aesthetics, stimulation etc.) (see Figure 4) (23).

#### 3.1.2. Participants and procedures

All children between 4 and 12 years old and their caregivers who had an appointment during the pilot study period were eligible for participation. Caregivers received an information folder together with their appointment letter prior to the appointment. The folder contained information on the Hospital Hero app as well as information on study participation. Caregivers could indicate if they were interested in study participation on the included reply card. They could also indicate if they wanted to participate in the online questionnaire, interview, or both. Caregivers and children could also download and play with the app without participating in study activities. Upon expressing interest in study participation, caregivers received an informed consent form. Informed consent was signed prior to all study activities. Due to capacity issues among postal delivery services as a result of the COVID-19 pandemic, not all eligible participants received the invitation letter. Consequently, initial response rates were low. To increase the response rate, we extended our recruitment strategy with a more personal approach, such that two research assistants invited eligible participants face-to-face at the outpatient clinic. Caregivers who expressed interest followed the same informed consent procedures as outlined above. Children and their caregivers had to meet the following inclusion criteria: (1) the child's age between 4 and 12 years old, (2) the child was able to speak, write and understand the Dutch language, (3) the caregiver was able to speak, write and/or understand the Dutch or English language. Caregivers who were not able to express themselves in Dutch or English were unable to participate.

Caregivers who expressed interest in participating in the interview were asked to fill out an observational booklet during the hospital visit, which was returned to the investigators (see Supplementary File 2). Participants were selected purposively to represent diversity in gender, age, medical background and whether they had to draw blood during the appointment. The sample size was determined by the principle of *a priori* and inductive thematic saturation which we expected to achieve with 20 children and their caregivers.

#### 3.1.3. Data collection

After the hospital visit, caregivers received an online questionnaire assessing use, user-experience and usability. In addition, demographic data were collected on gender, age, frequency of hospital visits within the past 24 months and whether the visit included a blood draw. The questionnaire was directed to and developed for children. Caregivers were invited to provide help with filling out the questionnaire if necessary. Children could indicate if they had received help with filling out the questionnaire.

##### 3.1.3.1. Usability

Usability was assessed using the System Usability Scale (SUS) (24). This is a generic instrument to measure the usability of a technology or service. It contains ten items rated on a 5-point Likert scale from 1 = “strongly disagree” to 5 = “strongly agree”. An additional free text field allowed for commenting on usability.

##### 3.1.3.2. User-experience

User-experience was assessed with the short version of the User-Experience Questionnaire (UEQ-S), which was made age-appropriate and tailored to the Hospital Hero app. The UEQ-S consists of eight items rated on a 7-point Likert scale from −3 and 3, with 0 as neutral) in the two dimensions of pragmatic and hedonic quality ((25). Responses to the UEQ-S items included an open text field to argue the response given. All the UEQ scales have a good to high reliability of.69 or higher (22).

We aimed to also assess user-experience with the Pick-A-Mood (PAM) tool, a cartoon-based pictorial instrument to measure self-reported mood states (26). A tablet was installed at different locations at the outpatient clinic on which, following instructions by the staff, children could select the pictogram that represented their mood at that moment. Due to the increased covid-related care burden on the hospital staff, the PAM data was not collected consistently enough to acquire reliable data and therefore disregarded from quantitative data analysis.

##### 3.1.3.3. Use

Use and user patterns were assessed based on the question “did you play with the app” in the online questionnaire and from interview and observation data.

##### 3.1.3.4. Qualitative data collection

Qualitative data was collected through semi-structured interviews. The interviews were held online due to covid-19 measures, using the videoconference software Microsoft Teams and were held within 5 days after the hospital appointment. In addition, caregivers were asked to fill in an observational booklet during the visit, as observations by the researchers were not possible to due to covid-19 restrictions. Semi-structured interviews were guided by a topic list, which was developed with a remedial educationalist and a pediatric nurse and followed the theoretical framework. The topic list consisted of main and probing questions and was tailored to the age of the participating child (see Supplementary File 3). Only the topic list for older children included temporal questions (e.g., when was that, how did you feel at that moment). An interview toolbox was developed containing screenshots of the app, photographs of the outpatient clinic and the PAM tool. These were used, if deemed helpful, to help retrieve memories and as conversation starters. Interviews were conducted by an experienced qualitative researcher and a remedial educationalist in training. After each interview, field notes were taken, including reflective notes of one's role.

#### 3.1.4. Data analysis and data handling

Quantitative data were analyzed using descriptive statistics on the SUS and UEQ-S scores. (SPSS version 25; IBM, Amonk, NY, United States). A-priori defined subgroup analyses were performed among subgroups for age (dichotomized into age 4 till 8 years and 9 till 12 years) and gender. Considering the fact that our evaluation was formative by design (i.e., gain understanding for improvements) instead of summative (i.e., to measure performance or specific end-points), we did not perform a power calculation. we did consider the size of the study population (20–25 appointments per day), an expected participation rate of 0.2 and recommendations by developers of the UEQ who recommend 51 and 70 participants for pragmatic and hedonic scale respectively (sampling confidence level 95%,margin of error = 0.01)(25). Responses to the online questionnaire were used to triangulate the qualitative findings (e.g., to find out how many participants indicated difficulties operating the app and to inform understanding of use and user feedback). Interviews were audio recorded and transcribed verbatim and anonymized. Qualitative data were analyzed following the Framework Method (27). Data were coded inductively and deductively, guided by constructs of the ISO norm but also leaving room for newly emerging codes (Atlas.ti, version 7.5.15). Codes were explored, recoded and used to identify categories (i.e., group of codes around similar and interrelated ideas or concepts). Categories were mapped and discussed, which resulted in the formulation of themes.

#### 3.1.5. Ethical considerations

The study was cleared for ethics by the Medical Ethical Review Committee of the Leiden University Medical Centre (No N20.199). Written informed consent to participate in this study was provided by the participants' legal guardian/next of kin. Children were verbally informed about participations in the study. Information was tailored to the age of the child.

### 3.2. Results pilot study

In total, 44 child-caregiver pairs expressed their interest in participating in the semi-structured interview. In the end, we included 21 child-caregiver pairs for the semi-structured interview and received the observational booklet from 31 caregivers. Children had diverse ages (range 4–11 years), gender and medical background (see Supplementary File 4). In addition, we sent out the online questionnaire to 71 child-caregiver pairs, of which 50 returned the questionnaire [four questionnaires were excluded because of missing data (i.e., >27 questions unanswered)]. The overall mean age was 8.2 years (SD 2.8), the majority was boy (*n* = 28; 61%) and most children had to draw blood during the appointment (*n* = 26; 57%) Children who did not need to draw blood had other physical examinations (e.g., ECG or cardiac ultrasound) or only a consultation.

**Figure 5:**
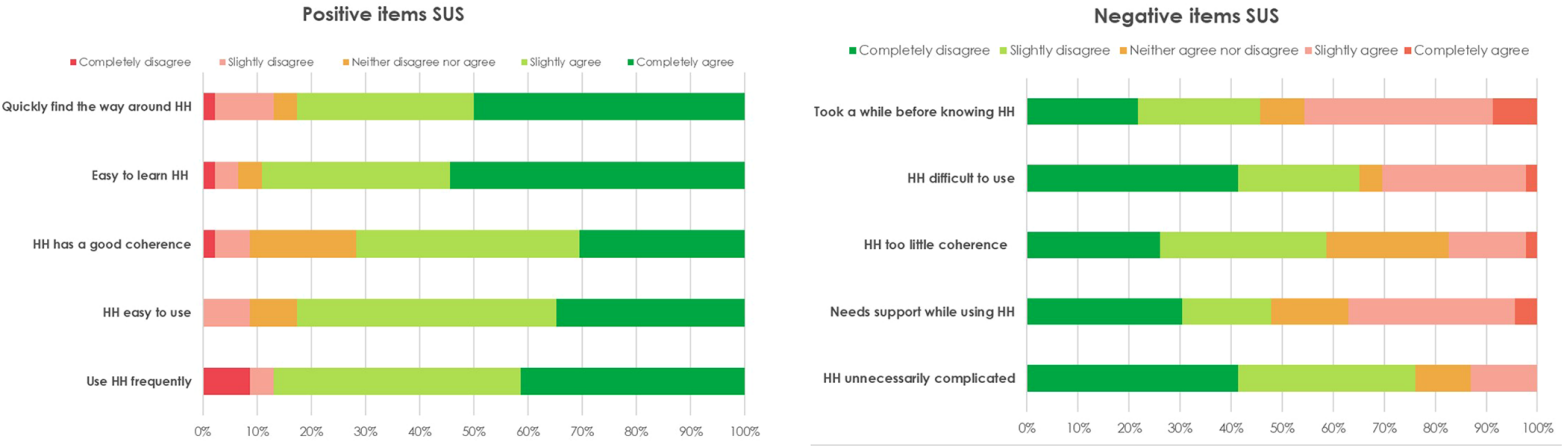
Results SUS item scores divided in negative and positive formulated items.

Five key themes were identified from our qualitative data, which were then triangulated and complemented with the quantitative descriptive data from the questionnaires. Each theme is described below, integrating both qualitative and quantitative data. Descriptive statistics for usability and user-experience are displayed in Table 2 and Figure 5.

**Table 2:** Descriptive statistics (*n* = 46) on overall score on the system usability scale (SUS) and user-experience short questionnaire (UEQ_S).

	Min	Max	mean ± SD
SUS	25	100	71.32 ± 17.80
UEQ_S	−.49	2.63	1.49 ± .95
Pragmatic	−2.50	3.00	1.31 ± 1.28
Hedonic	−2.75	3.00	1.49 ± 1.37

SUS, System Usability Scale (SUS); UEQ_S, User Experience Questionnaire Short (UEQ_S).

#### 3.2.1. Theme 1: User friendliness.

Overall, children and their caregivers were positive about the user-friendliness and usability of the app. This observation was supported by the SUS total score from the questionnaire, which averaged 71.32 (SD = 17.80). Benchmark comparison indicated an overall “good” usability. 87% of all respondents indicated that they would use the app more frequently (completely agree or slightly agree).

However, younger children and their caregivers mentioned that the child sometimes needed help from their parents, especially in understanding how to scan the QR codes and navigate between the different screens. Children aged 4 or 5 years also asked for help with reading the texts. The problems with difficulties of use were also observed in the SUS item scores. Almost 35% of all children needed support while using the HH app (completely agree or slightly agree). Comparison of the SUS score showed that older children had a statistically significantly higher overall SUS score (73.13 ± 15.92). However, two-way ANOVA analysis of the overall SUS score on age and gender only showed a main effect for gender [F(1) = 3.89, *p* = .05] but not for age ([F(1) = 0.36, *p* = .57] nor the interaction between the effect of gender and age [F(1) = 0.23, *p* = .64]. When asked if the usability problems hindered the playing, some children indicated that they enjoyed the interaction with their parents, whereas other children wanted complete control.

*“I did not see that [explanation screens on QR scanning], but I did notice that mom had to point out certain things to me. Also, she searched with me, but I thought: ‘this is for children.”*” – Boy, 10 years old.

Caregivers added that they enjoyed the interaction moments with their child and the app.

#### 3.2.2. Theme 2: Coherence and power of storytelling

SUS scores on items on coherence (HH has good coherence and HH has little coherence) showed that around 40% of the children found that the app lacked good coherence. The lack of good coherence was also observed in the qualitative data, which suggested that children did not find the storyline explicit enough in the app. Also, while children enjoyed choosing their favorite animal in the beginning and “searching for the QR codes, only one child referred to elements of the storyline such as going on a safari with their favorite animal. Two children suggested another way to strengthen the storyline in the app, for example, by having a more central character that they repeatedly meet in the app:

*“Maybe there should be a more adventurous story put into it. As if, for example, you come to a village of monkeys. And then maybe you come across the owner of a mine, who will say: ‘my worker is gone’. Then you must find the QR code that belongs to him. You get it?”* – Boy, 10 years old.

Next to the storyline in the app, children and caregivers missed that some healthcare professionals did not ask about the game or seem to be involved. This detracted somewhat from the overall experience and suggests that it is important that healthcare professionals engage in creating an immersive imaginative safari story. Likewise, healthcare professionals that did engage with the story facilitated engagement and improved the overall experience.

*“No, the doctor did not help. The blood sampling nurse, she was super excited about HH and searched everywhere with her. But the doctor did not, he said sort of: ‘well, good luck searching’”* – Mother of 8 year old girl.

When asked how to improve the healthcare professionals' engagement, caregivers indicated that healthcare professionals could for example give hints and show interest in the child's animal collection. Two caregivers also suggested adding the possibility for the child to add in how they feel before the appointment or to write down questions for the doctor, all to facilitate interaction between child and doctor.

#### 3.2.3. Theme 3: Motivation and reward

All interviewed children indicated they were motivated to engage with the app. They were attracted by the design and liked the vibrant colors and animals. Some children above ten years old found the app a bit “ childish” due to the drawing style of the animals and the difficulty level of the animal searching game. They wanted more challenging games such as puzzles, riddles, assignments, or animal quiz. They could imagine it to be fun for younger children. A comparison of scores of the UEQ_S - hedonic scale showed that boys and younger children scored statistically higher than girls and older children, respectively, indicating that they found the app more appealing.

When asked if they had collected animals, all children indicated that they had collected some or all animals. The majority of the children however did not understand that the animals indicated as silhouettes in the animal collection could only be collected during the next visit. Children felt demotivated, “disappointed” and confused as they could not collect all animals.

Children did like reward elements such as the virtual confetti rain, and the funny GIF when all animals were collected. They suggested to add additional reward elements such as winning clothes to dress the animal, money, tokens, points or extra assignments.

*“But also that if you played a game, you receive money. And with that money you can buy clothes for your animals”* – Boy, 9 years old.

#### 3.2.4. Theme 4: fit with the real hospital journey

An important recurring theme throughout all interviews was the fit with the hospital journey. This could be seen in two different ways. First, the alignment of the true hospital journey with the journey portrayed in the app (map) should be as optimal as possible. The map used a fixed number and sequence of steps. However, not all children needed to draw blood and thus engage with the step. Some children did not mind, however, two children mentioned that this step was confusing and caused more anxiety.

Second, the alignment in terms of timing is important for the overall experience. According to the participants, there should be sufficient time to search for all the animals and enough time to devote attention to playing instead of waiting. One child explained:

*“I did not watch the videos because I thought, I did not know how much time I had. So I just went ahead and did the fun part, seek the animals.” - Boy, 10 years old*.

Also, it should be clear to children and caregivers where the children can engage with the app. Caregivers were sometimes unsure if children were “allowed” to play in the consultation room or during the weighing and measuring.

#### 3.2.5. Theme 5: Procedural comfort

Though not part of the initial research question and not explicitly asked, most of the caregivers indicated that the children were more at ease as they were distracted from the waiting. The children were focused on the animal search and caregivers observed positive emotions such as enjoyment and pride upon finding another animal. Two caregivers reported that they enjoyed seeing their child searching with another child. One mother noted that seeing her child be at ease helped her to relax.

However, the distraction was only temporary, as illustrated by the following quote:

 “*The distraction was due to the QR codes. She was just seeking through the blood sample room. But we found the code the moment she was poked. The nurse said, ‘look how mom is scanning, look what will happen’. At this moment, the monkey came out. So it gets the pressure off of her. But the needle needs to stay in her arm for a while to take three vials of blood. So yes, within a second, she started screaming ‘take the needle out, the needle must come out, I feel it, it is hurting!’* ”– Mother of 6 year old daughter.

While almost all respondents experienced some distraction, one mother indicated that the app disrupted her standard routine used to help her child cope:

“*..if I am honest, we had a kind of a routine and this interrupted it a little bit. She is comfortable in her own expectation pattern. Besides that, she is also a bit shy with unfamiliar people, which I really noticed in the waiting room with all the people sitting there.”* – Mother of 6 year old daughter.

Regarding the preparation part, it should be noted that due to covid-related postal delays, most respondents had not downloaded the app at home but in the hospital. Hence, they had not engaged with the app at home nor used it as a preparation tool. Also, caregivers did not know about the animation video explaining the blood drawing procedure.

When asked if caregivers would think it would help the children prepare, caregivers indicated that they thought it was helpful. Those who visited the hospital regularly added that they wished they could have had the app for the first hospital visit.

### 3.3. Recommendations

Children and caregivers were asked for suggestions how to improve the app during the interview and with an open question in the online survey. Suggestions were clustered into four domains: age differentiation, usability, tailored and immersive journey and others. All recommendations are depicted in Box 1.

BOX 1 **Design recommendations by children and parents.**

**Age differentiation**

• Content differentiation for younger children (between 4 and 7 years old) and older children (between 7 and 12 years old)
• Spoken text for younger children
• Quiz or challenges for older children
• More reward elements (e.g. reward tokens to “ purchase” clothes for the animals’.

**Usability**

• Cue that all animals are collected and step is completed
• Notification that animal can be found in another room
• Interactive instruction screens
• More clear instructions on how to use and navigate app for younger children
• Automatic play of video’s

**Tailored and immersive journey**

• possibility to select route or procedure
• possibility to indicate first visit or follow-up visit
• Storyline stronger integrated within app and physical world

**Other recommendations**

• Distraction during blood drawing procedure
• Include more smaller games
• Make QR codes more appealing (e.g. integrate the animal theme)

## 4. Discussion

This paper described the development and evaluation of an eHealth solution to reduce preprocedural stress and anxiety following a PD service design approach, which resulted in the Hospital Hero app. The Hospital Hero app guides children in their journey to and through the hospital. By helping children prepare in their familiar environment in a fun and appealing way through gamification, the Hospital Hero app engages and empowers children and informs them about medical procedures. Children can continue their journey at the hospital by searching and collecting animals through QR code scanning, creating an overall more positive and engaging experience. The pilot study of the Hospital Hero app in practice showed that the app is positively evaluated on usability and user-experience and is considered feasible.

We adopted a PD approach, involving children, caregivers, healthcare professionals, and other stakeholders throughout the process. This approach allowed us to develop a novel intervention that is child-centered, takes into account the perspective of child, caregiver and healthcare professional and integrates state-of-the-art knowledge from all stakeholders (i.e., knowledge on procedural comfort, children, education, psychological preparation). Hence, by involving multiple stakeholders, we were able to develop an intervention focusing on optimizing the experience for children and caregivers while working with available resources and requiring minimal efforts from healthcare professionals.

### 4.1. Patient-centered outcomes

This study demonstrated how a PD service design approach can help to develop interventions to improve pediatric patient experience by taking the experiences of the child, caregiver and healthcare provider as a starting point. Using this approach, we were able to identify critical moments in a child's journey that add to the build-up of stress and anxiety, find ways how to alleviate these moment and benefit the overall patient experience. Increasingly, medical academics acknowledge that overall patient experience is important in creating more beneficial patient-centered outcomes, such as satisfaction of care, self-efficacy and trust in healthcare, and clinical outcomes, such as recovery time (28). As such, patient experiences are increasingly adopted in pediatric and adult care as important indicators for the quality of healthcare. The Hospital Hero app improves the overall experience by taking the patient's experience journey as a starting point and by intervening in the potentially anxiety-heightening moments. These moments do not only involve interactions between child and healthcare professionals, but also entering the hospital building and waiting in the weighing room, but are critical for the overall experience.

### 4.2. Effective mechanisms in the Hospital Hero app

Evaluation of the Hospital Hero in practice helped us to gain in-depth insight in the user experiences and interactions and identify effective mechanisms. The pilot study showed that children specifically enjoyed searching for animals, and this took their attention away from waiting or negative emotions and feelings (in the case of children who experience preprocedural stress and anxiety). With the animal search, children are given a task (searching for and collecting animals) that they actively engage in. These more active ways of distraction are powerful as they actively divert the focus from negative emotions and the (anticipated) procedure (29). These findings correspond with studies evaluating the use of VR in which children are actively distracted by playing games (30). Moreover, the animal search requires problem-solving skills which help to empower children and give them a feeling of autonomy (I can do it myself) if performed successfully (31). By regularly changing the animals and hiding places, children can endeavor on the search each time they visit the hospital. Based on previous research, the connection of the game world with the physical world (i.e., physical QR codes as game tokens) promises to be especially powerful in transferring the child's acquired coping skills to the real-world and is absent in preparations platforms such as “Xploro” (14, 32).

The feeling of autonomy and control is strengthened by the fact that the app is child-centered and directed towards the child (i.e., speaking to the child instead of about the child) without requiring active involvement from a caregiver. However, parental involvement and more specifically, their level of stress and anxiety is pivotal in reducing (pre)procedural distress in children. Parental involvement has even been considered an important moderator of pain perception and stress response (13). Research has shown that children experience more distress and more intense pain during medical procedures if their caregivers show certain distress-related behaviors, such as (unconscious) projection of own psychological distress, provision of reassurance, empathic comments and excessive explanations and apologies to their children, whereas use of humor and talking about topics other than the medical procedure are associated with increases in a child's adaptive coping (33, 34). The Hospital Hero can support caregivers by acting as a conversation tool and introduce stress-reducing terminology (i.e., comfort talk) that they can use. Future developments should explore how to enhance parental guidance within the Hospital Hero app and take the moderating effects of parental involvement into consideration.

Finally, three design opportunities can be identified: (1) tailored journey, (2) differentiation and (3) timing of engagement. Whereas the current Hospital Hero app is built of fixed steps and focused on blood drawing only, future developments should be focused on the possibility to tailor a child's journey to the specific procedures involved. Second, the app's content should be differentiated according to age and medical experience of children. Children between four and seven generally liked the app, found it engaging and fitted their problem-solving skill levels. To appeal to older children it is important to differentiate in age and include more challenging activities (35). Use of, for example AI, could be useful in tailoring and deriving the content to the knowledge level of each individual child, as applied to the “Xploro” platform (14).

Third, the timing of when and how long to engage with the app should fit with the particular hospital visit. For example, there should be no additional distraction during the consultation and children should be distracted long enough during the waiting to reach optimal effects on anxiety levels.

### 4.4. Implications for practice and research

Our study demonstrated multiple critical moments outside of the consultation room where stress and anxiety are present (e.g., anticipation of pain, new environment), some which have been described before (36). Future comfort care strategies should look beyond the hospital walls and explore how to diminish the build-up of stress. This asks for nurses and doctors to be more mindful of the entire experience journey of their patients (e.g., the child's preparations, expectations, and previous experiences).

While this study took the patient journey of children visiting the Willem-Alexander Children's hospital as starting point, the Hospital Hero application was built in a modular way. This allows the app to be tailored to other hospitals and to be extended with additional preparation modules (e.g., preparation game for weighing and measuring or spirometry). A modular build is important to create a product that transcends the medical silos and prevents the introduction of an app for every specialism or medical procedure. In addition, the modular build supports scale-up potentials and the adaptation to other healthcare settings where children undergo (medical)procedures and experience stress and anxiety, such as primary care practices, dentist practices and within vaccination programs (37–39).

### 4.5. Limitations

Both studies had some methodological challenges. Firs, we were restricted in our data collection methods due to covid −19 measures. In the first study, we were unable to test the working prototype with children and caregivers at the outpatient clinic and set up a simulated hospital instead. Early testing in the outpatient clinic could have improved our understanding of the user experience earlier in the process (e.g., preferences during consultation). In the second study, we were unable to perform observations at the hospital and interviews were held online, a necessity many researchers reverted to to continue data collection during the pandemic. Although a review of online interviews and focus groups showed that the online setting does not compromise the quality of the data (40), it is unknown if this also goes for interviews with children. Nonetheless, we undertook multiple measures to ensure good quality data. We used an interview toolkit with photos and pictorial images to facilitate the conversation, had caregivers fill in an observation booklet during the appointment, which was used during the interview, and made use of screenshots of the app. Also, to minimize the burden on the healthcare professionals we decided not to collect their experiences in the evaluation. By evaluating the use of the Hospital Hero in practice we were however able to shed light on potential implementation issues (e.g., engagement of all healthcare providers, management of QR codes, reaching and activating caregivers to download the app) that need to be addressed in future research and implementation.

Second, due to covid related postal capacity problems, not all participants received the invitation letter and did not download the app. Consequently, the onboarding process (being informed and activated to download the app) could not be properly evaluated. Interviews did give insight into how the onboarding process could be improved. Third, children and caregivers who participated in the study may have been more positive about the application overall. We tried to minimize this by setting out a broad recruitment strategy, approaching caregivers that showed no initial interest in the application in the face-to-face recruitment and emphasizing the importance of hearing all opinions during the interview.

### 4.6. Future directions

Real-world evaluation allowed us to identify important implementation issues that should be addressed in the future, like how to activate caregivers to download the app at home and how to engage all staff in the safari story. Hence, follow-up research should focus on how best to implement the Hospital Hero in practice, setting up an implementation strategy and evaluating the implementation efforts. Implementation and scaling efforts should be directed at being minimally disruptive to daily practice, creating stakeholder support and demonstrating impact for all stakeholders involved.

Finally, we demonstrated that the app was evaluated positively by children and caregivers and has the potential to diminish stress and anxiety. This pilot evaluation study should thus be considered a first step in the eHealth evaluation cycle (41). Future research should be directed at assessing the app's impact on patient outcomes (i.e., stress and anxiety, procedural knowledge, patient satisfaction), healthcare professionals (i.e., satisfaction) and healthcare as a whole (i.e., cost-benefit). The impact method guided by the Quadruple Aim framework could be a useful way to evaluate impact on the short and long term for all stakeholders (42).

## 5. Conclusion

Applying a PD approach, we developed a novel child-centered eHealth intervention that was evaluated positively on use and user experience and has the potential to reduce preprocedural stress and anxiety by focusing on all anxiety-heightening moments before and during an outpatient visit. The real-world pilot setting helped us to identify three important design improvement opportunities. It also helped us to understand the interaction between the child, caregiver and the Hospital Hero app and provided in-depth insight into implementation issues to address in future research and implementation. As such, the Hospital Hero app can be considered an important addition to the toolbox that healthcare professionals use in their comfort care.

## Data Availability

The raw data supporting the conclusions of this article will be made available by the authors, without undue reservation.
