# Supplementary material for: How to use participatory design to develop an eHealth intervention to reduce preprocedural stress and anxiety among children visiting the hospital: The Hospital Hero app multi-study and pilot report
Source: Front Pediatr. 2023 Feb 14;11:1132639. doi: 10.3389/fped.2023.1132639 (PMC9971988; doi:10.3389/fped.2023.1132639)
Supplement: Supplementary file 2 [file Datasheet2.pdf]

Observatie lijst

Gebruikersonderzoek
Hospital Hero app

Datum:

Tijd:

Code deelnemer:

Instructie

Bij een gebruikers onderzoek kijkt normaal gesproken een onderzoeker mee tijdens het gebruik van een app. Door COVID-19 is dat op dit moment helaas niet mogelijk. Daarom willen we u als ouder vragen om in deze test de rol van de onderzoeker over te nemen. U kunt in deze observatielijst bijhouden op welke manier uw kind de app gebruikt gedurende het hele ziekenhuis bezoek.

Dit boekje is ingedeeld per ruimte waar u tijdens het bezoek zult komen. Links staat iedere keer een plattegrond van de ruimte afgebeeld. Daar kunt u aankruisen op welke plekken uw kind zich bevindt tijdens het gebruik van de app. Op de rechter pagina kunt u drie app-gebruik momenten beschrijven. U kunt de aangekruiste plek met het tijdstip verbinden zoals in het voorbeeld op deze pagina.

Alvast hartelijk dank voor uw bijdrage aan dit gebruikers onderzoek!

⦿ **Tijdstip**	15:43
Aandachtspunten	Opmerkingen/voorbeelden
**Wat valt u op?**	Ze moest lachen
**Hoe gebruikt uw kind de app?**	Ze houdt de app voor zich en kijkt ernaar
**Wat doet uw kind?**	QR codes zoeken
**Hoe voelt uw kind zich?**	enthousiast, blij (omcirkeld), ontspannen, verveeld, verdrietig, bezorgd, boos

Noteer het tijdstip

Noteer wat u op dat moment opviel.

Noteer op welke manier uw kind op dat moment de app gebruikte. Beschrijf letterlijk wat u ziet gebeuren, niet wat u denkt dat er gebeurt.

Noteer wat uw kind op dat moment precies deed.

Omcirkel de emotie die volgens u het gevoel van uw kind op dat moment het beste weerspiegelt.

Grote wachtruimte

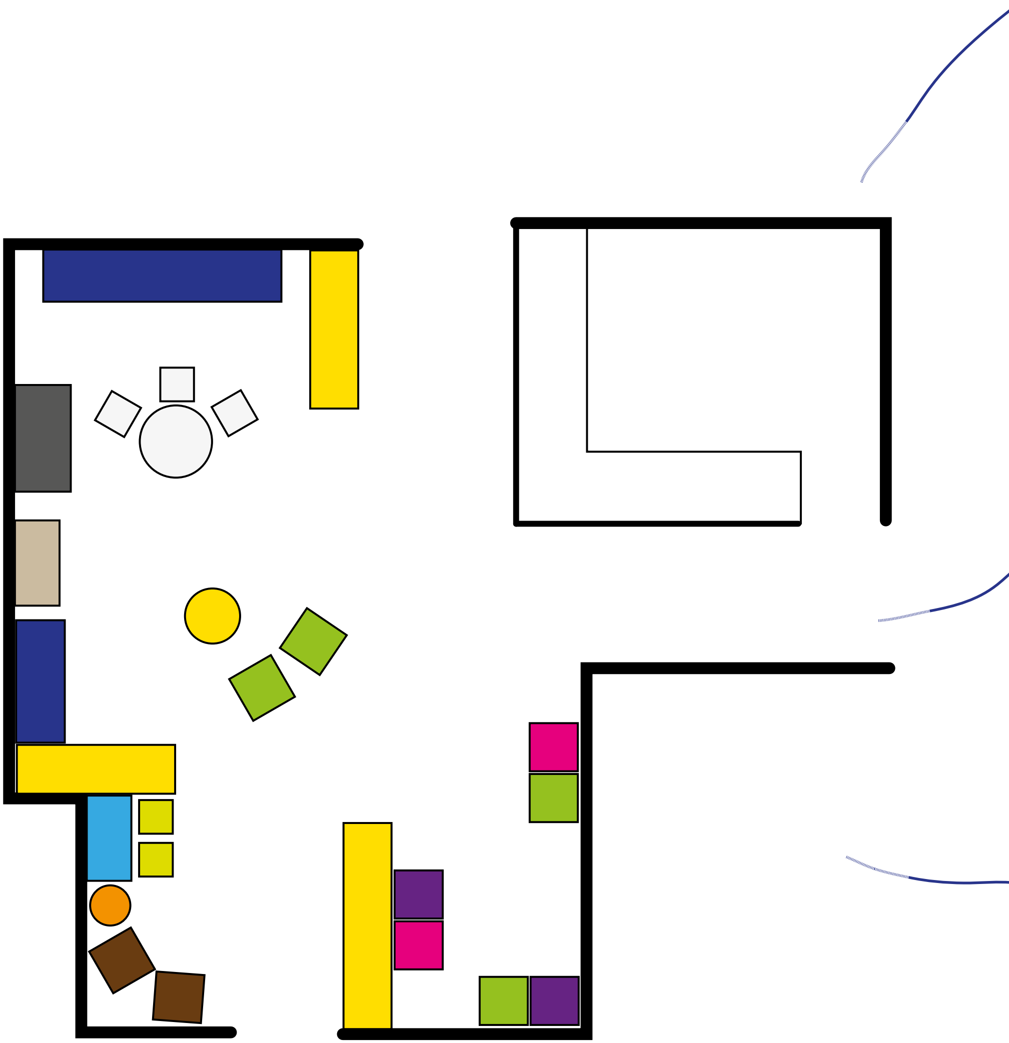

Tijdstip	
Aandachtspunten	Opmerkingen/voorbeelden
**Wat valt u op?**	
**Hoe gebruikt uw kind de app?**	
**Wat doet uw kind?**	
**Hoe voelt uw kind zich?**	enthousiast, blij, ontspannen, verveeld, verdrietig, bezorgd, boos

Tijdstip	
Aandachtspunten	Opmerkingen/voorbeelden
**Wat valt u op?**	
**Hoe gebruikt uw kind de app?**	
**Wat doet uw kind?**	
**Hoe voelt uw kind zich?**	enthousiast, blij, ontspannen, verveeld, verdrietig, bezorgd, boos

Tijdstip	
Aandachtspunten	Opmerkingen/voorbeelden
**Wat valt u op?**	
**Hoe gebruikt uw kind de app?**	
**Wat doet uw kind?**	
**Hoe voelt uw kind zich?**	enthousiast, blij, ontspannen, verveeld, verdrietig, bezorgd, boos

Meten en wegen

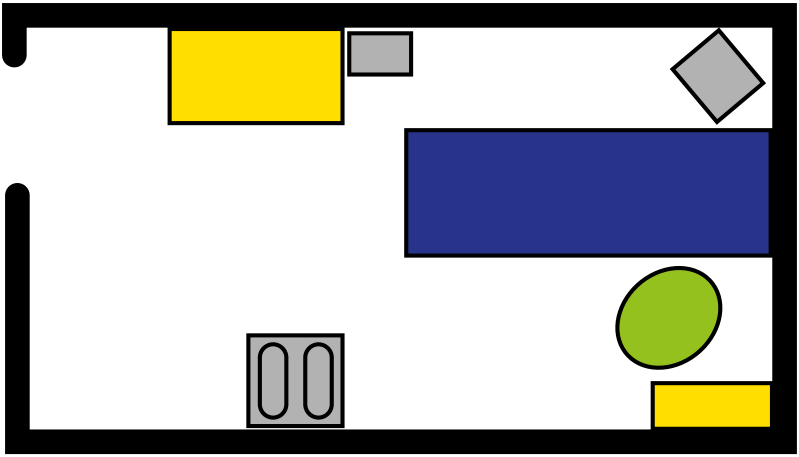

**Tijdstip**	
Aandachtspunten	Opmerkingen/voorbeelden
**Wat valt u op?**	
**Hoe gebruikt uw kind de app?**	
**Wat doet uw kind?**	
**Hoe voelt uw kind zich?**	enthousiast blij ontspannen verveeld verdrietig bezorgd boos

**Tijdstip**	
Aandachtspunten	Opmerkingen/voorbeelden
**Wat valt u op?**	
**Hoe gebruikt uw kind de app?**	
**Wat doet uw kind?**	
**Hoe voelt uw kind zich?**	enthousiast blij ontspannen verveeld verdrietig bezorgd boos

**Tijdstip**	
Aandachtspunten	Opmerkingen/voorbeelden
**Wat valt u op?**	
**Hoe gebruikt uw kind de app?**	
**Wat doet uw kind?**	
**Hoe voelt uw kind zich?**	enthousiast blij ontspannen verveeld verdrietig bezorgd boos

Arts

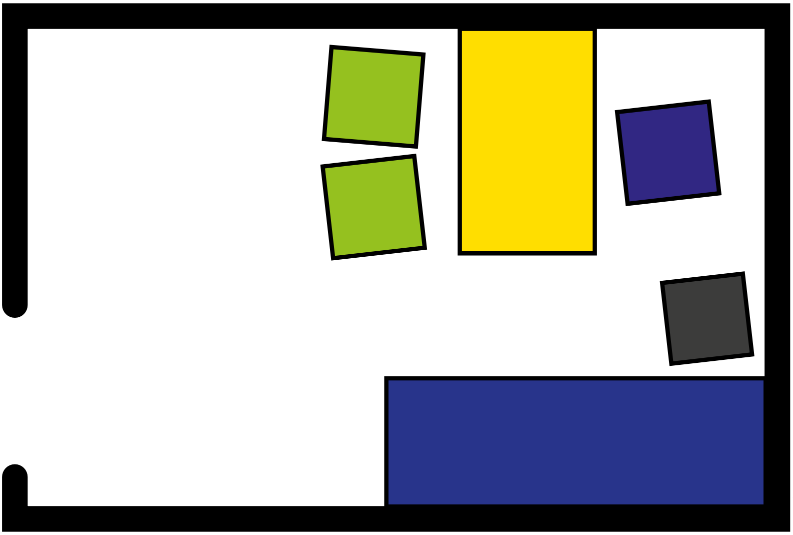

**Tijdstip**	
Aandachtspunten	Opmerkingen/voorbeelden
**Wat valt u op?**	
**Hoe gebruikt uw kind de app?**	
**Wat doet uw kind?**	
**Hoe voelt uw kind zich?**	enthousiast blij ontspannen verveeld verdrietig bezorgd boos

**Tijdstip**	
Aandachtspunten	Opmerkingen/voorbeelden
**Wat valt u op?**	
**Hoe gebruikt uw kind de app?**	
**Wat doet uw kind?**	
**Hoe voelt uw kind zich?**	enthousiast blij ontspannen verveeld verdrietig bezorgd boos

**Tijdstip**	
Aandachtspunten	Opmerkingen/voorbeelden
**Wat valt u op?**	
**Hoe gebruikt uw kind de app?**	
**Wat doet uw kind?**	
**Hoe voelt uw kind zich?**	enthousiast blij ontspannen verveeld verdrietig bezorgd boos

Kleine wachtruimte

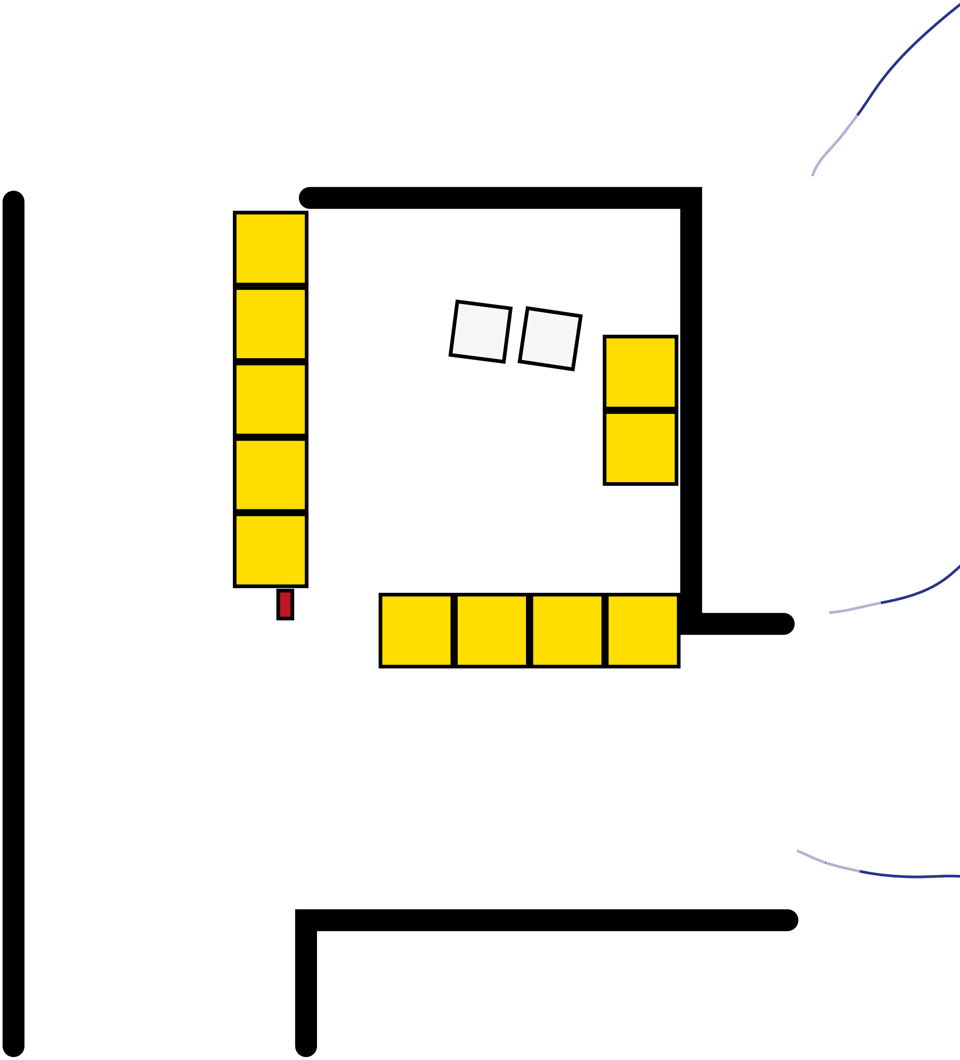

◉ **Tijdstip**	
Aandachtspunten	Opmerkingen/voorbeelden
**Wat valt u op?**	
**Hoe gebruikt uw kind de app?**	
**Wat doet uw kind?**	
**Hoe voelt uw kind zich?**	enthousiast blij ontspannen verveeld verdrietig bezorgd boos

◉ **Tijdstip**	
Aandachtspunten	Opmerkingen/voorbeelden
**Wat valt u op?**	
**Hoe gebruikt uw kind de app?**	
**Wat doet uw kind?**	
**Hoe voelt uw kind zich?**	enthousiast blij ontspannen verveeld verdrietig bezorgd boos

◉ **Tijdstip**	
Aandachtspunten	Opmerkingen/voorbeelden
**Wat valt u op?**	
**Hoe gebruikt uw kind de app?**	
**Wat doet uw kind?**	
**Hoe voelt uw kind zich?**	enthousiast blij ontspannen verveeld verdrietig bezorgd boos

Kinderpost

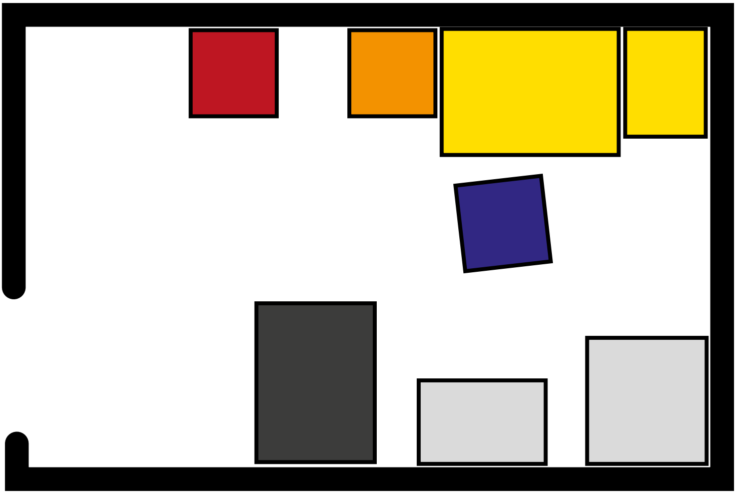

**Tijdstip**	
Aandachtspunten	Opmerkingen/voorbeelden
**Wat valt u op?**	
**Hoe gebruikt uw kind de app?**	
**Wat doet uw kind?**	
**Hoe voelt uw kind zich?**	enthousiast · blij · ontspannen · verveeld · verdrietig · bezorgd · boos

**Tijdstip**	
Aandachtspunten	Opmerkingen/voorbeelden
**Wat valt u op?**	
**Hoe gebruikt uw kind de app?**	
**Wat doet uw kind?**	
**Hoe voelt uw kind zich?**	enthousiast · blij · ontspannen · verveeld · verdrietig · bezorgd · boos

**Tijdstip**	
Aandachtspunten	Opmerkingen/voorbeelden
**Wat valt u op?**	
**Hoe gebruikt uw kind de app?**	
**Wat doet uw kind?**	
**Hoe voelt uw kind zich?**	enthousiast · blij · ontspannen · verveeld · verdrietig · bezorgd · boos
