# Supplementary material for: How to use participatory design to develop an eHealth intervention to reduce preprocedural stress and anxiety among children visiting the hospital: The Hospital Hero app multi-study and pilot report
Source: Front Pediatr. 2023 Feb 14;11:1132639. doi: 10.3389/fped.2023.1132639 (PMC9971988; doi:10.3389/fped.2023.1132639)
Supplement: Supplementary file 3 [file Datasheet3.pdf]

Interviews Pilot study of the Hospital Hero app - 4 to 8 years

Introduction

- Introduce, explain briefly ('ask questions about the Hospital Hero app, 'wonder what you think', purpose of interview. 20 min with child, then with father/mother

- Explain what happens to information that child gives -> help people who make the app to make it better, to be able to help more children

- Do you mind if I record the interview? [written informed consent given by parent prior to interview]

Opening interview

- Questions age, class

- Do you remember the last time you were in the hospital?
  probe: with whom, what have you done?

- Asking about specific times of visit (waiting room at the doctor's, having blood drawn, waiting for that, going home)
  probe: what did the room look like, what did you do there? Have you used/played with the HH app?

Core topics interview

- Do you remember what you did with the app in the [waiting room/ at the doctor's office/ in the children's mail room]?
  probe: what, where, with whom, which animals?

- What do you think of the app? Why?
-
- When you heard about the app, what did you think it was for?

- Was it always clear what you could do in the app? Do you understand all the things in the app?

- Was it always clear how to do things in the app?
  Probe: catching/collecting animals, back to the road map, choosing your favorite animal

- Were you able to use the app at any time? (At home, in the waiting rooms, at the doctor's, children's post)

Ease of use/attractiveness

- What did you think of using the app? Which things were difficult and which were easy?

- Were there things in the app that you liked or liked? Which things?

Design suggestions

- What ideas do you have that would make the HH app even better?

Closing

- Indicate that the pre-agreed time has passed, so round it off
- Reflecting on how the conversation went (even if it wasn't easy, name it, but then with a compliment).
- Closing question: Would you like to tell me something about the HH app you find important to tell me?
- Thank you for telling me so much!

-

Interviews Pilot study of the Hospital Hero app - 9 to 12 years old

Introduction

- Introduce, explain briefly ('ask questions about the Hospital Hero app, 'wonder what you think', purpose of interview. 20 min with child, then with father/mother

- Explain what happens to information that child gives -> help people who make the app to make it better, more children can help

- Do you agree if the interview is recorded?

- Make it clear that we want to know the child's opinion, there are no right or wrong answers

Initiate interview

- Questions age, class

- Do you remember the last time you were in the hospital?
    probe: with whom, what have you done?

Core interview

- How did you experience the hospital visit? How did you feel (in the run-up to the visit, in the waiting rooms, at the doctor's office, at the nursery)?

- How did you use the HH app?
    probe: What, when, where, and with whom? (Use photos and observation booklet if useful to retrieve memories, follow up on parent observations)

- What did you think of the HH app (at home and in hospital)? Why?

Expectations

- When you got the app, what did you think it was for?

- After using it, what do you think the app is for?

Clarity/Functionality

- Was it always clear what you could do in the app? Do you understand all the things in the app?

- Was it always clear how to do things in the app?
    probe: catching/collecting animals, back to the road map, choosing your favorite animal

- Were you able to use the app at any time? (At home, in the waiting rooms, at the doctor's, children's post)

Ease of use/attractiveness

- What did you think of using the app? Which things were difficult and which were easy?

- Were there things in the app that you liked or liked? Which things?

Fitness

- Have you been to the hospital before? If so, do you think your visit to the hospital has been changed by the HH app? And what exactly has changed?

The intention of the HH app is to better prepare for the visit to the hospital and to make the visit itself more fun.

- Did the HH app prepare you well/better for the hospital visit? What things in the app caused this?

- Has the HH app made your visit to the hospital more fun/enjoyable? What things in the app caused this?

Design suggestions

- What ideas do you have that would make the HH app even better?

Closing

- Indicate that the pre-agreed time has passed, so round it off

- Reflecting on how the conversation went (even if it wasn't easy, name it, but then with a compliment).

- Closing question: Would you like to tell me something about the HH app we did not mention?

- Thank you for telling me so much!
