# Supplementary material for: How to use participatory design to develop an eHealth intervention to reduce preprocedural stress and anxiety among children visiting the hospital: The Hospital Hero app multi-study and pilot report
Source: Front Pediatr. 2023 Feb 14;11:1132639. doi: 10.3389/fped.2023.1132639 (PMC9971988; doi:10.3389/fped.2023.1132639)
Supplement: Supplementary file 4 [file Datasheet4.pdf]

Multimedia Additional File 4. Characteristics of participants in online interviews (Study 2)

	Study ID	Gender	Age	Blood Drawn during appointment
**1**	004-1	Girl	8	Yes
**2**	004-2*	Girl	6	Yes
**3**	011	Boy	9	unknown
**4**	016	Boy	8	unknown
**5**	018	Girl	6	unknown
**6**	019	Boy	8	unknown
**7**	025	Girl	6	Yes
**8**	026*	Boy	10	Yes
**9**	028	Girl	7	No
**10**	031	Boy	10	Yes
**11**	043	Boy	8	unknown
**12**	045	Girl	4	No
**13**	049	Boy	7	Yes
**14**	053	Boy	4	Yes
**15**	058	Girl	5	unknown
**16**	061	Boy	10	Yes
**17**	062	Boy	7	Yes
**18**	063	Girl	8	unknown
**19**	067	Boy	10	No
**20**	080	Girl	6	unknown
**21**	089	Girl	11	unknown

B: boy; G:girl
