# Supplementary material for: How to use participatory design to develop an eHealth intervention to reduce preprocedural stress and anxiety among children visiting the hospital: The Hospital Hero app multi-study and pilot report
Source: Front Pediatr. 2023 Feb 14;11:1132639. doi: 10.3389/fped.2023.1132639 (PMC9971988; doi:10.3389/fped.2023.1132639)
Supplement: Supplementary file 1 [file Datasheet1.pdf]

Ideal Hospital Hero Journey

Preparation at home	Upon arrival	Big waiting room	Weighing and Measuring	Consultation room	Small waiting room	Blood drawing procedure	Departing	At home

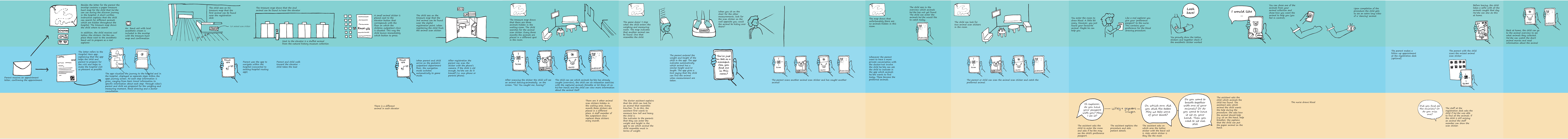

Assumption: Parent downloads the app before going to the hospital.

Assumption: Parent prepares for the hospital visit.

Assumption: Parent prepares his/her child at home before going to the hospital.

Assumption: Looking for animals using a treasure map is a fun activity to do in the hospital

Assumption: the treasure map is usefull to find your way through the hospital

Assumptions: the app helps to navigate through the hospital

Assumption: Finding a scan sticker is fun for the child

Assumption: child understand that the small animal sticker indicates the pediatric department.

Assumption: the child understand from the desing of the sticker that it can be scanned with the phone's camera

Assumption: looking for scan stickers (animals) is fun

Assumption: three scan stickers is enough to be busy

Assumption: the treasure hunt stimulates the child to go searching for animals

Assumption: upon arrival the parent has enough time and ease of mind to hand over the phone to the child for the animal hunt.

Assumption: A GIF of a dancing animal is a nice reward

Assumption: Some children prefer to read more information about the animal

Assumption: Children prefer to see an overview of all the animals they collected

Assumption: weighing and measuring becomes more fun when the child knows he/she will recieve an animal as reward

Assumption: parents join in the game

Assumption: searching for a sticker is equally nice from the chair (as walking around)

Assumptions: scanning the room for the sticker helps to be distracted from the procedure

Assumptions: the procedure is less stressfull or scary when child is distracted

Assumption: parents prefer that the child is occupied themselves.

Assumption: the child enjoys to choose two additional animals that he/she wants to collect

Assumption: it is fun collecting an animal which your have chosen uit yourself.

Assumption: two scan stickers is enough in this room

Assumption: The pain passport fits logically within the patient journey

Assumption: Children have a stronger feeling of being on control because they can decide wich animals will support them and how.

Assumption: Breathing together with the selected animal helps the child relax

Assumption: looking at an virtual animal sitting on the child's hand helps the child to sit still and be relaxed

Assumption: An extra long video of a dancing animal is a nice reward
